# Transphyseal ACL reconstruction resulted in small incidence of tibial physeal bars at 1-year follow-up

**DOI:** 10.1016/j.jor.2025.03.007

**Published:** 2025-03-18

**Authors:** Mahsa Sarrami, Payam Tarassoli, Yoong Ping Lim, Alex Nicholls

**Affiliations:** a Sydney Orthopaedic Research Institute, St Leonards, Australia; b The Children's Hospital at Westmead, Australia; c Charles Darwin University, Casuarina, NT, Australia

**Keywords:** Transphyseal ACL reconstruction, Growth arrest, Physeal bar formation

## Abstract

**Introduction:**

Transphyseal anterior cruciate ligament reconstruction (ACLR) is associated with a risk of angular limb deformity and limb length discrepancy particularly when there is a significant period of growth remaining.

Previous studies on the effects of transphyseal ACLR have used inconsistent methodology to estimate bone age, and therefore subjects may have underestimated the effect on the physis.

This study sought to evaluate the incidence of iatrogenic physeal bar formation following ACLR using high-resolution MRI and a validated bone age atlas.

**Methods:**

A prospective series of patients undergoing transphyseal ACLR at a single institute, with high resolution 3T MRIs at 12 months, were included. MRI-validated knee bone age atlas was then used to exclude patients with evidence of physiological physeal closure. The remaining skeletally immature MRI scans were appraised by two independent reviewers for the presence of physeal bar formation adjacent to transphyseal ACL tunnels.

**Results:**

From 142 patients undergoing transphyseal ACLR with post-operative MRI, 94 patients were found to exhibit evidence of complete closure of either the tibial or femoral physis and were excluded. 48 patients (38 male – mean age 14.1; 10 female – mean age 13.9) meeting inclusion criteria were included. Mean tibial tunnel diameter was 8.2 mm (SD ± 0.81) and mean femoral tunnel diameter was 7.9 mm (SD ± 0.88). There were two cases (4.2 % of total) of physeal bar formation in the proximal tibia in males (aged 14.2 and 14.7 years) with otherwise open physes. No leg length discrepancy was observed for these patients at a 12 months. No cases of femoral physeal bar formation were identified in our cohort.

**Conclusion:**

High resolution 3T MRI scan performed 12 months after transphyseal ACL reconstruction demonstrated 4.2 % incidence of tibial physeal bars and no femoral physeal bars. Neither of the cases physeal bar cases exhibited angular deformity or limb length discrepancy. Central physiological closure of the tibial physis was commonly seen in this age group and may be easily confused with physeal bar formation.

## 1 Introduction

Anterior cruciate ligament (ACL) tears are an increasingly common injury in the skeletally immature adolescent^1^ perhaps owing to increased participation in organized, year-round competitive sport..^2^^,^^3^ Whilst bracing and non-operative management remain acceptable treatment strategies, surgical management more reliably restores stability, thereby reducing the risk of secondary chondral and meniscal damage and allowing higher rates of return to sport.^4^^,^^5^

However, as ACL reconstruction (ACLR) in patients with open physis carries a risk of iatrogenic disruption a wide variety of surgical techniques have been developed to mitigate physeal risk. For patients with less than 3 years of growth remaining, transphyseal ACLR is one such technique favoured by many surgeons as it strikes a balance to reduce the risk of re-rupture by placing tunnels in more anatomical positions. Given transphyseal ACLR has graft tunnel with less axial tilt angle (i.e. less oblique), there will be reduced surgical cutting impact across the physeal surface area. A recent review of the literature found an overall low rate of growth disturbance (2.9 %) in transphyseal ACLR, however the authors noted that the included studies expressed a large degree of heterogeneity, particularly concerning the determination of bone age, thereby making it difficult to interpret the relationship.^6^ Chotel described three ways in which the growth plate can be injured as a result of ACL reconstruction surgery^7^^,^^8^: Type A, with a physeal bar of bone forming across the growth plate causing a tether “arrest”; Type B, resulting from an increased metabolic activity and overgrowth; and type C, as a result of the tension and tenoepiphysiodesis effect. Type A, or physeal bar formation, is defined as a bridge that restricts and may cause asymmetric growth arrest around the epiphysis.^9^

Concerning the assessment of bone age, published literature on transphyseal paediatric ACLR uses a variety of physiological classifications to assess patients’ level of skeletal maturity, such as Tanner Staging.^10^, ^11^, ^12^ However radiographic staging, predominantly using hand radiographs,^13^ is considered the gold standard.^14^ Physiological classifications can be inaccurate due to gender, ethnicity and nutritional variation amongst diverse patient populations.^15^ This variability may have underestimated the true impact of transphyseal tunnels morbidity on the growing physis, because some patients who have completed growth may be included in previous studies which use only choronological age and not radiographic criteria.

Pennock et al.^16^ have developed a Knee Magnetic Resonance Imaging (MRI) bone age atlas to assess skeletal maturity and bone age based on the reproducible sequence of skeletal ossification of the patella, tibia, fibula and femur.

Assessment of physeal injury may be visible in the form of a consequent clinical deformity, or the presence of “bony bar” formation across the physis adjacent to ACLR tunnels. A bony bar may act as a bridge, potentially causing growth arrest by tethering. Greater accessibility and advances in MRI scan resolution have resulted in the ability to confirm skeletal immaturity and then review post-operative MRIs to assess the relationship between tunnel size, position and growth disturbance.^17^^,^^18^

This study sought to accurately assess the incidence of Chotel type A growth injury and physeal bar formation in patients undergoing paediatric transphyseal ACLR by utlising high resolution 3T MRI, and a contemporary method of determining physeal maturity.

Our aim was to determine the incidence of physeal bar formation at 12 months following paediatric transphyseal ACLR. Our secondary aim was to assess the relationship between tunnel size (as a ratio of physis surface area) and risk of physeal bar formation. We hypothesized a low rate of growth disturbance in transphyseal ACLR pediatric cohort who remained skeletally immature based on MRI.

## 2 Materials and methods

A single institute prospective patient series was collected between 2015 and 2022 at our medical institution. Inclusion criteria was all paediatric patients who had undergone transphyseal ACL reconstruction utilizing hamstring autografts. Each patient underwent an anatomical single-bundle ACLR, which fellowship-trained orthopaedic surgeons performed via arthroscopy. The semitendinosus tendon was harvested as the hamstring autograft and an adjustable suspensory was used to fixate the graft within the knee joint. Femoral tunnel placement was done via an anteromedial portal and an inside-out reaming method. The tibia tunnel was created using the outside ACLR footprint guide. The graft and the suspensory fixation device were deployed from the lateral femoral condyle; the other end of the graft was then fixed on the anteromedial tibia using a cortical button with the reconstructed knee being fully extended.

At the 12-month mark post-surgery, high-resolution 3T MRI scans were performed. The MRI protocol,^19^ developed exclusively at our research institute, employed 0.5 mm slices without separations. The high-resolution 3D turbo spin echo proton density sequence imaging technique provided accurate assessment and clarity in reviewing the anatomical structures surrounding the ACL graft tunnels.

To ensure a comprehensive evaluation of patient skeletal maturity and assess the impact of the procedure, we employed a validated knee bone age atlas developed by Meza et al..^20^ The MRI bone age assessment was conducted both prior to surgery and again 12 months after the surgical intervention. Patients exhibiting significant signs of physiological physeal closure indicative of skeletal maturity were excluded from the study.

Patients displaying partial physiological physeal closure were considered eligible for inclusion if more than two-thirds of the growth plate (physis) remained open, and the area of closure did not extend within 1.5 cm of the locations of the ACL graft tunnels.

The MRI scans of the remaining skeletally immature patients were assessed by two independent fellowship trained orthopaedic surgeons. Using the MRI bone age atlas,^16^ the femoral, tibial and fibular physes were categorized as: open physis, partial physeal closure or complete physeal closure. Furthermore, harris growth arrest lines were recorded if present as a separate potential indicator in altered endochondral ossification.

The reviewers objective was to identify any evidence of physeal bar formation adjacent to the transphyseal ACL tunnels, in order to determine the relationship between the surgical procedure and the physeal changes in the knee region.

**Figs. 1:**
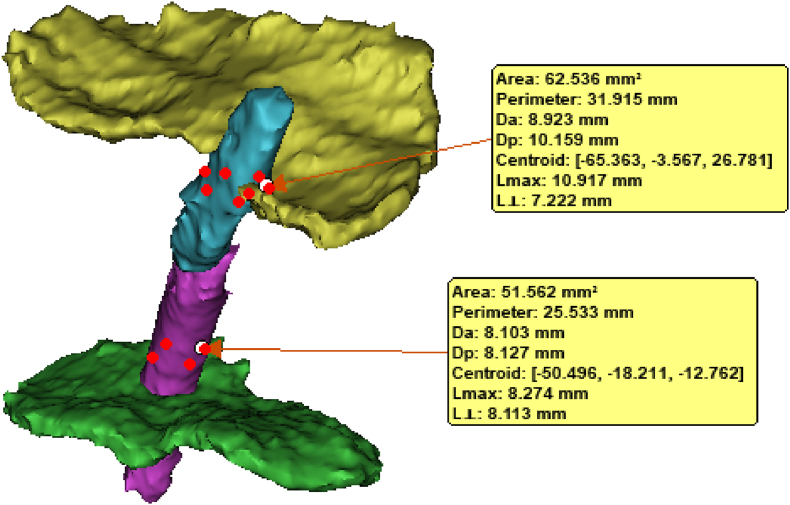
3D reconstruction using the highly validated MRI scans.

Bilateral leg length discrepancy was determined by the bilateral difference between hip and ankle joint centres imaged using 2-dimensional x-ray in the frontal anatomic plane. 3D models of the ACL tunnel, femur physeal and tibia physeal were generated using 3D processing software (Mimic Medical 21.0 and 3-matic, Materialise, Belgium). The models were created via manual segmentation by a biomedical engineer. Measurement plane was defined by best-fitting multiple points placed on each physis; corresponding tunnel slice and physeal were then projected on the measurement plane to quantify the tunnel to growth plane ratio. 3D volumetric analysis of tunnel size and physeal surface area demonstrated tunnel size as a percentage of growth plate surface area [Fig. 1]*.*

## 3 Results

142 patients undergoing both transphyseal ACL reconstruction and 12 month post-operative MRI were identified. 94 patients were found to exhibit evidence of complete closure of either the tibial or femoral physis and were excluded. There were 48 patients (38 male, 10 female) meeting inclusion criteria.

The mean age at time of surgery was 14.0 years (range 9.8–16.4). The mean age at time of surgery for females was 13.9 years (SD ± 0.7, range 12.5–14.6) and for males was 14.1 years (SD ± 1.6, range 9.8–16.4). Bone age, measured on pre-operative MRI and at 12 months post-operatively did not differ significantly from chronological age [Table 1].

**Table 1:** – Patient chronological age vs bone age.

	Male (n = 38)	Female (n = 10)	All (n = 48)
Chronological age: Mean (SD)	14.0 years (±1.6)	13.9 years (±0.7)	14.1 years (±1.6)
Bone age based on MRI atlas	14.0 (±1.5)	13.3 (±1.3)	13.9 (±1.4)
Paired T-test	0.626	0.772	0.557

**Fig. 2:**
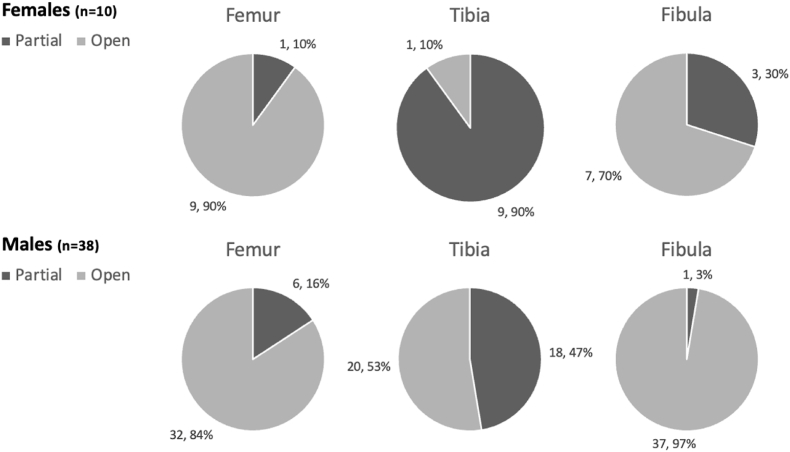
– Pie charts demonstrating the distribution of 48 patients with open physis and partial physeal closure of growth plates of the included patients 12 months Post ACL Reconstruction based on MRI Scan.

All included patients had open or partially open physes. Fig. 2 illustrates that all cases of physeal closures were excluded, leaving only patients who are still growing at 12 months after surgery was performed. Tibial physeal closure occurs earlier than the femur or the fibula.

### 3.1 Growth Complications

**Fig. 3:**
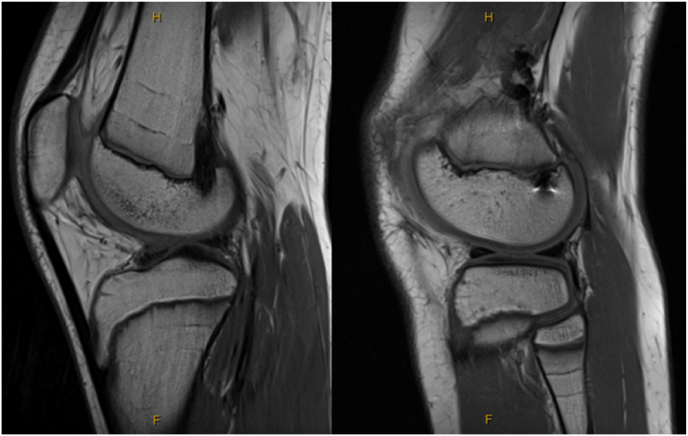
– Skeletally immature patient post operative MRI image showing Harris growth arrest lines.

Harris growth arrest lines were present in 42 % of tibias, 73 % of femurs and 31 % fibulas. [Fig. 3], representing a transient slowdown in endochondral ossification at the growth plate.^21^ The presence of Harris growth arrest lines (HGA lines) in the fibula, may signify the global effect on the region as compared to direct impact from drilling for tunnels.

**Fig. 4:**
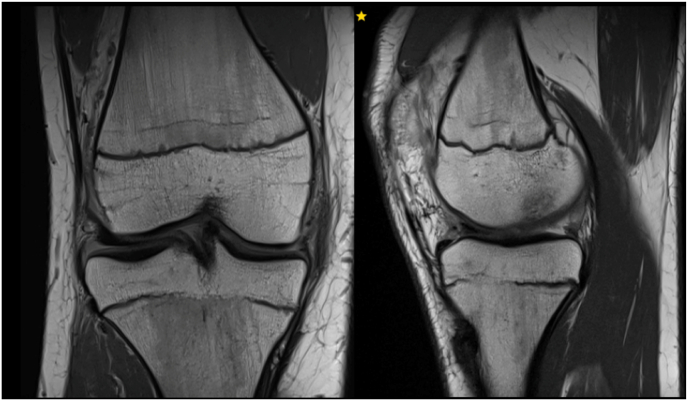
MRI scan showing physeal bar formation in the tibia.

We identified 2 cases of tibial physeal bar formation in two males (ages at date of surgery 14.2 and 14.7) both of whom had otherwise completely open physes [Fig. 4]. No leg length discrepancy or alignment disruption was observed for these two patients at a 12 month post-operative long leg alignment scan. This gave an incidence of 4.2 % in our studied cohort. There were no cases of femoral bar formation.

**Fig. 5:**
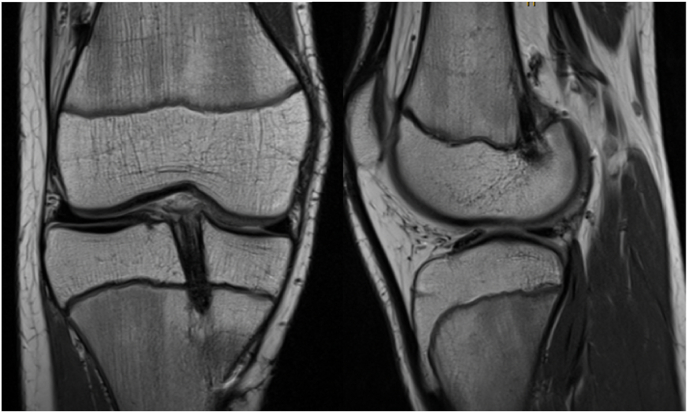
– MRI scan showing no physeal bar formation adjacent to tibial or femoral graft.

We identified one case of clinically important reduced leg length of 2.1 cm (1.2 cm shorter on operated femur and 0.9 cm on operated tibia). This patient had no sign of physeal bar formation on review of MRI 12 months post operation [Fig. 5]*.* This patient underwent contralateral epiphysiodesis.

MRI measurements demonstrated that the mean tibial tunnel size was 8.2 mm (SD ± 0.81 mm) and femoral tunnel size 7.9 mm (SD ± 0.88 mm). Tibial growth plate tunnel size as a percentage of growth plate surface area was 2.2 % (SD ± 1.1) and femoral tunnel 2.7 % (SD ± 1.3) [Table 2]. These tunnel ratios were consistent with previous literature.^22^ There was no significant relationship between physeal bar formation and tunnel size to physis ratio [Table 2].

**Table 2:** – Comparing growth-plate ratios across different patient groups.

	Tibial Tunnel Size: Physis Ratio	Femoral Tunnel Size: Physis Ratio
Tenoepiphysiodesis Group (n = 1)	1.94 %	1.7 %
Physeal Bar Group (n = 2)	1.7 % (STD 0.33)	2.0 % (STD 0.23)
All Others (n = 45)	2.2 % (STD 1.1)	2.7 % (STD 1.3)
Physeal Bar vs All Others (p-value)	0.228	0.076

## 4 Discussion

The most important finding of this study was that the incidence of iatrogenic physeal bar formation is relatively low, even in patients with reliably determined physeal immaturity and highly sensitive 3T MRI measured 12 months after surgery.

The findings of this study therefore provide further insight to the incidence of physeal bar formation in paediatric ACLR patients and its potential implications on clinical outcomes.

By ensuring that only children with open growth plates around the knee were included, the study actively reduced confounding variables to provide more accurate data regarding physeal bar formation. Gicquel et al.^23^ found 14 cases (20 % incidence) of physeal bar formation, however did not include a bone age tool in their criteria. Yoo et al.^24^ identified 5 cases (11.6 % incidence) of physeal bar formation and used hand bone age x-ray as their bone age tool. Our study adopted rigorous inclusion criteria, utilizing advanced imaging techniques and a validated knee bone age atlas to distinguish between physiological physeal closure and iatrogenic growth plate injury. This emphasis on accurate patient selection for the study means the incidence of visible physeal damage is highly accurate, primarily because skeletally mature patients have been excluded.

In agreement with our study hypothesis, only two cases (4.2 % incidence, a low disturbance rate) of physeal bar formation were identified in the tibial region. The authors believe that the appearance of the bony bridges directly adjacent to the transphyseal ACL tunnel should be considered iatrogenic, unless proven otherwise. Perhaps futures cases of recognised tibial bar formation could have contemporaneous contralateral knee MRI scan for comparison of the physiological physeal appearance.

Notably, no physeal bars were detected in the femoral region. The peripheral location of the femoral ACLR tunnel is located closely to the perichondrial ring of La Croix which has been previously reported as a structure that may be associated with growth abnormality when breached.^25^ Hence this absence of femoral physeal bars is reassuring. The relatively low incidence of physeal bar formation in this study corroborates previous research that suggests growth-related problems after transphyseal ACL reconstruction are rare. An interesting observation in this study is that neither of the two cases with tibial physeal bars displayed clinical consequences such as angular deformities or limb length discrepancies during the 12-month long leg alignment scans. This suggests that while physeal bars may form in a small percentage of cases, these occurrences may not necessarily lead to functional issues or limb deformity in the short term. However, it is crucial to underscore that the long-term impact remains uncertain, and further research with extended follow-up periods and alignment surveillance is necessary to determine whether these growth disturbances will remain asymptomatic.

The single case involving leg length discrepancy did not demonstrated any MRI evidence of physeal bar formation or injury. Pre-surgical EOS scan in this patient did show a small leg length discrepancy in this child on review of anatomical length (12 mm), however, this was certainly exacerbated by the ACLR surgery overtime (21 mm). This raises the issue of whether Chotel Type B growth disturbance (tenoepiphysiodesis effect) may play a significant role in generating observed growth disturbance in these patients. Further studies are required with similarly strict inclusion criteria in order to assess the incidence of the tenoepiphysiodesis effect on limb growth and alignment after ACLR.

The study's secondary aim was to investigate the relationship between tunnel size, expressed as a ratio of physis surface area, and the risk of physeal bar formation. While this specific aspect did not yield significant findings and was comparable to other literature,^17^ it is an important avenue for further research. Assessing whether the size of the surgical tunnels plays a role in the development of physeal bars may help guide surgical decisions and minimize the risk of iatrogenic growth disturbances.

There is a lack of literature review on presence of HGA lines post ACLR on MRI. A recent systematic review of 100 studies only found 3 papers commenting on HGA lines with only one paper reviewing it on MRI.^26^ Gicquel et al., reviewed 100 MRIs and described growth arrest as deviation from Harris line, but no mention of prevalence.^23^ We found that the majority of our cases (72 %), exhibited HGA lines in femur. The reduced prevalence of HGA in tibia could represent the earlier physiological closure of this physis in relation to femur. Similarly the presence of HGA in the fibula could indicate the global effect of the knee as a cause and not the tunnel. Larson et al.,^27^ found 4 of 29 cases (14 %) to exhibit HGA lines after review of radiographs. We found increased sensitivity of MRI in confirming presence of HGA lines, in keeping with literature on use of MRI with assessment of growth arrest.

The study reported a notably low incidence of physeal bars, bolstered by a robust radiographic approach for patient selection, but certain limitations warrant acknowledgment. One key limitation is the absence of universal pre-operative EOS scans or hand X-rays for all patients. These imaging modalities, which are widely used to assess skeletal maturity, were not uniformly available in our patient cohort. As a result, the study may not have captured the full spectrum of growth-related changes that could occur in response to the surgical procedure. Furthermore, the relatively few cases of physeal bar formation identified in this study indicate that it may be underpowered to detect smaller effects or subtle relationships between tunnel size and growth disturbances. Additional research with larger sample sizes might help shed further light on these potential associations. Although this study has a reasonably high sample size compared to the similar studies listed by the review study by Patil et al.,^6^ any non-statistical significant results should be interpreted cautiously due to the possibility of a type II error.

Our study highlights the importance of stringent inclusion criteria when assessing growth in paediatric patients. To truly understand the impact of surgical interventions on a developing musculoskeletal system, it is imperative to ensure that the study population consists solely of children who are still actively growing. While this study reported a low incidence of physeal bars, the risk is not negligible. Thus, clinicians and researchers alike must exercise caution, judicious post-surgical surveillance and employ rigorous patient selection criteria when working with this patient cohort. Paediatric ACL reconstruction remains a dynamic field, and further studies are necessary to refine our knowledge and ultimately improve patient outcomes.

## 5 Conclusion

The present study advances our understanding of iatrogenic physeal bar formation subsequent to transphyseal ACLR in paediatric patients who remain skeletally immature using high-resolution knee MRI. There was no case of femoral physeal bar formation identified and there was a 4.2 % incidence of iatrogenic tibial physeal bars. The tenoepiphysiodesis effect of the ACLR graft crossing femoral and tibial physis may significantly affect limb growth and alignment at longer follow-ups. The few cases of physeal formation within this study cannot properly examine the relationship between tunnel size and growth disturbances.

## Ethical approval

The study design was approved by the Human Research Ethics Committee of Northern Sydney Local Health District (NSLHD reference: RESP/17/110).

**Consent to Publish:** Participant provided written consent for publication of images.

**Informed consent**: Informed consent was obtained from all individual participants included in the study. If the participant was not of legal age to provide independent consent, both parental consent and child assent were obtained prior to data collection.

## Author contribution

Dr Mahsa Sarrami: Data Collection, Dr. Sarrami contributed to the collection of clinical data, including the patient's medical history, physical examination findings, and initial diagnostic tests. Literature Review, Dr. Sarrami conducted an extensive literature review to provide background information and context for the case report, Manuscript Writing: Dr. Sarrami was primarily responsible for first draft of the case report, Writing – review & editing: She critically reviewed and edited the manuscript, incorporating feedback from the co-author and reviewersDr Payam Tarassoli, Data Collection: Assisted in second review and contributed to the collection of clinical data, including the patient's medical history, physical examination findings, and initial diagnostic tests. Writing – review & editing: critically reviewed and edited the manuscript, providing feedback, Dr Yoong Lim, Data Collection. Was involved in 3D analysis of the data collected and contributed to the manscript through writing the methods and results related to this section, DrAlex Nicholls, Conceptualization: Dr. Nicholls was responsible for conceiving the idea for the project and outlining the initial framework.- Leadership: He advised throughout the process and encouraged and provided direction Writing – review & editing: Dr Nicholls collaborated closely with Dr Sarrami to revise and refine the manuscript, ensuring clarity and coherence.- Final Approval: Dr. Nicholls provided final approval for the version of the manuscript submitted for publication.

## Funding

- No funding received from any source for this project.

Flowchart 1. Representing patient selection.

## Conflict of interest

Dr Mahsa Sarrami (MD, Masters of Surgery) – no conflict of interest.

Dr Payam Tarassoli (MD) – no conflict of interest.

Yoong Ping Lim – associate editor of J. Orthopaedic Research Surg.

Alex Nicholls – no conflict of interest.
